# Cortical CD200–CD200R and CD47–SIRPα expression is associated with multiple sclerosis pathology

**DOI:** 10.1093/braincomms/fcae264

**Published:** 2024-08-07

**Authors:** Aletta M R van den Bosch, Dennis Wever, Pleun Schonewille, Sabine L Schuller, Joost Smolders, Jörg Hamann, Inge Huitinga

**Affiliations:** Neuroimmunology Research Group, Netherlands Institute for Neuroscience, Amsterdam, 1105 BA, The Netherlands; Neuroimmunology Research Group, Netherlands Institute for Neuroscience, Amsterdam, 1105 BA, The Netherlands; Neuroimmunology Research Group, Netherlands Institute for Neuroscience, Amsterdam, 1105 BA, The Netherlands; Neuroimmunology Research Group, Netherlands Institute for Neuroscience, Amsterdam, 1105 BA, The Netherlands; Neuroimmunology Research Group, Netherlands Institute for Neuroscience, Amsterdam, 1105 BA, The Netherlands; Department of Neurology, MS Center ErasMS, Erasmus Medical Center, Rotterdam, 3015 GD, The Netherlands; Department of Immunology, MS Center ErasMS, Erasmus Medical Center, Rotterdam, 3015 GD, The Netherlands; Neuroimmunology Research Group, Netherlands Institute for Neuroscience, Amsterdam, 1105 BA, The Netherlands; Amsterdam Institute for Immunology and Infectious Diseases, Amsterdam University Medical Center, Amsterdam, 1105 AZ, The Netherlands; Neuroimmunology Research Group, Netherlands Institute for Neuroscience, Amsterdam, 1105 BA, The Netherlands; Swammerdam Institute for Life Sciences, University of Amsterdam, Amsterdam, 1054 BE, The Netherlands

**Keywords:** multiple sclerosis, immune inhibition, normal-appearing grey matter

## Abstract

Control of microglia activity through CD200–CD200R and CD47–SIRPα interactions has been implicated in brain homeostasis. Here, we assessed CD200, CD47, CD200R and SIRPα expression with qPCR and immunohistochemistry in multiple sclerosis (MS) normal-appearing cortical grey matter (NAGM), normal-appearing white matter (NAWM), cortical grey matter (GM) lesions and perilesional GM, and compared this to control GM and white matter (WM), to investigate possible altered control of microglia in MS. In MS NAGM, CD200 expression is lower compared with control GM, specifically in cortical layers 1 and 2, and CD200 expression in NAGM negatively correlates with the cortical lesion rate. Interestingly, NAGM and NAWM CD200 expression is positively correlated, and NAGM CD200 expression negatively correlates with the proportion of active and mixed WM lesions. In GM lesions, CD200 and CD47 expressions are lower compared with NAGM and perilesional GM. CD200R expression is lower in MS NAGM, whereas SIRPα was increased in and around GM lesions. Taken together, our data indicate that CD200 and CD47 play a role in GM MS lesion formation and progression, respectively, and that targeting CD200 pathways may offer therapeutic avenues to mitigate MS pathology in both WM and GM.

## Teaser

Van den Bosch *et al*. report that in multiple sclerosis post-mortem human brain tissue, CD200 expression in normal-appearing grey matter is lower compared with controls, negatively correlates with cortical lesion load, and CD200 and CD47 expressions are reduced in cortical lesions and perilesional regions, suggesting a role in multiple sclerosis pathology.

## Graphical Abstract

**Graphical Abstract:**
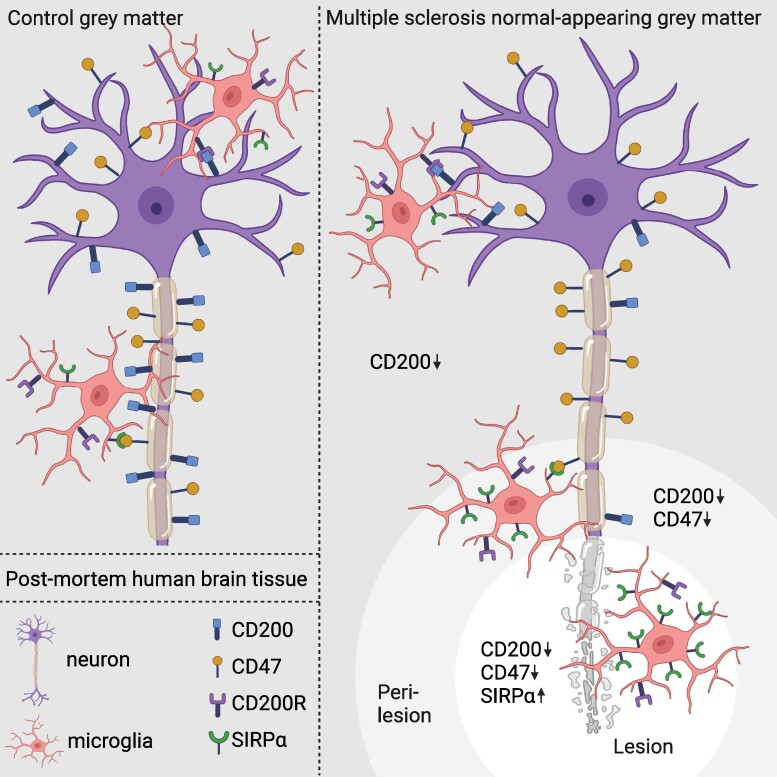

## Introduction

Multiple sclerosis (MS) is a chronic inflammatory disorder characterized by focal demyelinating lesions and neuroaxonal damage across the central nervous system (CNS).^1,2^ Within cortical normal-appearing grey matter (NAGM) of people with MS, diffuse neurodegeneration, microglia activation and macrophage infiltration are found, with variability observed among individuals.^3^ In MS NAGM compared with healthy grey matter (GM), microglia exhibit upregulated gene expression associated with iron accumulation and inflammation,^4^ increased density and altered morphology.^5^ Large areas of the cortical GM can be affected by MS, and the majority of patients have cortical GM lesions at autopsy.^6-10^ Based on their location in the cortex, GM lesions are classified as subpial, intracortical and leukocortical lesions.^11^ GM lesions and perilesional GM demonstrate increased apoptotic neuron counts and reduced neuronal density, contributing to the pathological characteristics of MS.^12,13^ Although the clinical consequences of GM demyelination are not yet fully understood, the extent of GM demyelination has been correlated with disability and cognitive impairment in people with MS.^9,14,15^

The interaction between neurons and microglia plays a role in microglia homeostasis, modulating microglia activity through both cell–surface interactions and soluble molecules.^16,17^ Combinations of inhibitory and stimulatory signals regulate microglia activity.^18^ In the healthy CNS, neurons actively maintain microglia in a quiescent, ramified state.^19^ CD200 and CD47 are immunoglobulin superfamily glycoproteins. CD200 is highly expressed by neurons, and to a lesser extent by oligodendrocytes, whereas CD47 is expressed by neurons, oligodendrocytes and astrocytes.^20^ Binding of CD200 and CD47 to their receptors CD200R and SIRPα, respectively, on microglia, results in maintaining microglia in a ramified, antiphagocytic state.^21-24^ Interestingly, in major depressive disorder, increased expression of CD200 and CD47 is thought to regulate an immune-suppressed microglial phenotype.^25^

In MS, neuron–microglia communication is disrupted.^17^ Previously, we found checkpoint molecules CD200 and CD47 are expressed lower in and around white matter (WM) lesions in post-mortem MS brain tissue compared with normal-appearing white matter (NAWM).^26,27^ In experimental autoimmune encephalomyelitis (EAE) mice, decreased CD200 levels manifest during the presymptomatic phase,^28^ and knock-out of CD200 elicited an accelerated microglial response, more severe pathology and a more rapid onset of EAE.^22,29,30^ Conversely, administering a CD200R agonist (CD200-Fc) during the chronic EAE phase mitigates disease severity, demyelination, axonal damage and microglial clustering within the CNS.^31^ Blocking CD47 *in vitro* promotes myelin phagocytosis,^32^ and intraperitoneal administration of antibodies against CD47 in late-stage EAE intensified disease severity and enhanced immune activation, leading to elevated immune cell proliferation and heightened production of inflammatory cytokines in spleen and lymph nodes.^32^ To date, it is not yet known what the expression levels of CD200, CD47, CD200R and SIRPα in NAGM, GM lesions and perilesional GM are, and their roles in lesion formation and disease severity are yet to be elucidated.

This study describes the expression of CD200, CD200R, CD47 and SIRPα in MS and control (NA)GM, GM lesions and perilesional GM. Furthermore, we correlated this to clinical and pathological hallmarks. We hypothesize that lower expression of CD200 and CD47 within the MS GM instigates an exaggerated microglial response, impeding remyelination processes, amplifying pathological cascades and thereby exacerbating the severity of the disease. Ultimately, the restoration or augmentation of CD200–CD200R and CD47–SIRPα interactions may emerge as a promising avenue for the potential treatment of MS.

## Materials and methods

### Donors and sample collection

Snap-frozen post-mortem brain tissue samples of the superior or medial temporal gyrus of *n* = 30 non-demented control donors and *n* = 34 MS donors, and fresh tissue samples of the lateral parietal sulcus and NAWM of the pyramid tract of 1 MS donor were provided by the Netherlands Brain Bank (www.brainbank.nl). All donors provided informed consent for brain autopsy and for the use of material and clinical data for research purposes in compliance with national ethics guidelines. MS pathology was confirmed by a certified neuropathologist. MS and control donors were matched for age, sex, post-mortem delay, pH of the CSF and weight of the brain, and the donor demographics are summarized in Table 1. For MS donors, disease duration was calculated as years between initial symptom onset and death. Ranked scores on three pathologically and clinically relevant dimensions of disease progression were previously calculated based on data accessible using the Netherlands Neurogenetics Database (NND).^33,34^ Lesion parameters were calculated as previously described.^8,35^ In standard locations of the brainstem, the reactive site load was calculated as the number of reactive sites identified, and the lesion load was calculated as the sum of all lesions present. The proportions of active, mixed, inactive and remyelinated lesions throughout the CNS were calculated by dividing the number of lesions of that lesion type by the sum of active, mixed, inactive and remyelinated lesions. The cortical lesion rate was calculated by dividing the number of cortical lesions present throughout the CNS by the number of tissue blocks containing the cortex.

**Table 1:** Donor demographics

Parameter	Control (*n* = 30)	MS (*n* = 32)	*P*-value
Age (years)	73.10 ± 12.26	70.26 ± 10.23	n.s.
Sex	22 F/8 M	16 F/16 M	n.s.
PMD (min)	396.50 ± 101.19	502.74 ± 100.20	1.2e−4
pH CSF	6.55 ± 0.41	6.45 ± 0.25	n.s.
Weight of the brain (g)	1242.19 ± 112.98	1201.38 ± 134.53	n.s.

CSF, cerebrospinal fluid; F, female; M, male; PMD, post-mortem delay.

### RNA isolation and qRT-PCR

For each tissue block, (NA)GM was dissected with a scalpel in the cryostat and two 20 μm sections were made. The (NA)GM was separated from (NA)WM, and was collected in 500 μl of Trisure (Bioline, London, UK). The tissue was dissociated by vortexing rigorously and the samples were stored at −80°C until RNA isolation. The samples were incubated with 200 μl chloroform for 2 min and spun down at 10 000 rpm for 10 min. The aqueous phase was collected, and an equal amount of cold isopropanol was added together with 1 μl glycogen. The samples were left to precipitate at −20°C for an hour and spun down at 10 000 rpm for 10 min. The supernatant was discarded, and the samples were air-dried. The pellet was reconstituted in 20 μl RNase-free water and incubated at 55°C for 10 min. RNA concentration and purity were measured on the NanoDrop (Thermo Fisher, Waltham, MA, USA).

Complementary DNA (cDNA) was synthesized with the Quantitect Reverse Transcription Kit (Qiagen, Hilden, Germany). Samples of 12 μl with 1000 ng RNA were incubated with 2 μl of gDNase at 42°C for 5 min and subsequently incubated with 6 μl of cDNA master mix at 42°C for 30 min and at 95°C for 3 min. cDNA samples were diluted in the proportion 1:20 in RNase-free water and stored at −20°C.

Quantitative reverse transcription PCR (qRT-PCR) was performed in duplo using the primers indicated in Table 2. The samples were diluted 1:1 with the SYBR Green PCR Master Mix (Applied Biosystems, Waltham, MA, USA) and 2 μM primer mix per reaction. For each gene of interest, the primer mix contained 2 μM forward primer and 2 μM reverse primer (Sigma-Aldrich, St. Louis, MO, USA). The QuantStudio PCR system 96-well apparatus (Thermo Fisher) was used for the amplification. For each reaction, the cycle threshold (CT) was assessed. Samples containing lesions as assessed with immunohistochemistry (IHC) (*n* = 11 MS samples) or samples where genes of interest were not detected with qPCR were excluded (*n* = 2 control, *n* = 6 MS samples). The average CT of each duplicate reaction was subtracted by the CT of the average of the two housekeeping genes (ΔCT). The ΔCT was subtracted by the average ΔCT of the control group (ΔΔCT) and the −ΔΔCT was calculated per gene per donor.

**Table 2:** qPCR primers

Primer	Forward sequence (5′–3′)	Reverse sequence (5′–3′)
*CD200*	CCAGGAAGCCCTCATTGTGA	TCTCGCTGAAGGTGACCATGT
*CD47*	ATGGAGCTCTAAACAAGTCCACTGT	TGTGAGACAGCATCACTCTTATCCAT
*CD200R1*	GAGCAATGGCACAGTGACTGTT	GTGGCAGGTCACGGTAGACA
*SIRPA*	GTCTGGAGCAGGCACTGA	GGACTCGCAGGTGAAGCT
*GAPDH*	TAGTCGCCGTGCCTACCAT	CCTGCTGCCTTCCTTGGA
*EEF1A1*	AAGCTGGAAGATGGCCCTAAA	AAGCGACCCAAAGGTGGAT

### Immunohistochemistry

Fresh tissue was fixed for 24 h in 4% paraformaldehyde (PFA) after autopsy, and tissue was submerged in 30% sucrose before being frozen at −80°C. Tissue sections of 8 µm thickness of the frontal gyrus or 20 µm thickness of the snap-frozen lateral parietal sulcus and pyramid tract were cut using a cryostat. Antibodies and their corresponding fixation buffers were selected as detailed in Table 3. For optical density (OD) analysis, dilution series were made to ensure that the DAB precipitation had not reached saturation and that the staining would develop in a controlled, time-dependant manner, which is necessary to distinguish subtle changes in protein expression. The OD values over the dilution series and the OD values of the chosen primary antibody over incubation time of DAB are shown in Supplementary Fig. 1. The sections used for different stainings were sequential, and all MS and control sections per staining were stained simultaneously. For fresh-frozen sections, sealed frozen sections were allowed to reach room temperature for 30–45 min and then fixed for 15 min using 4% PFA or 10 min using ice-cold acetone. Acetone-fixed tissue was air-dried for 40 min. For free-floating immunofluorescent (IF) stainings, the sections were washed with PBS. Subsequently, the sections were treated with 3% hydrogen peroxide (H_2_O_2_) in phosphate-buffered saline (PBS) + 0.5% TritonX for 20 min before being incubated with blocking buffer (PBS + 10% normal horse serum + 1% bovine serum albumin + 0.5% TritonX) for 1 h to block aspecific binding. Primary antibodies were incubated overnight at 4°C. For all DAB stainings and for IF staining of axons [neurofilament heavy chain (NFH)], biotinylated secondary antibodies 1:400 were incubated for 1 h and incubated with avidin–biotin complex (ABC; Vector Laboratories, Newark, CA, USA) 1:800 in PBS for 45 min. For CD200R, the staining was enhanced with biotinylated tyramide 1:7500 + 0.001% H_2_O_2_ in 0.05 M borate-buffered saline for 10 min and incubated with ABC 1:800 in PBS for 45 min. For all DAB-visualized stainings, the sections were incubated with 3-3′-diaminobenzidin (DAB) for 10 min to visualize the staining, followed by counterstaining with haematoxylin and dehydration using increasing ethanol concentrations and xylene. All IF stainings were incubated with the appropriate fluorophore-conjugated antibodies. For nuclei staining, DAPI (4',6-diamidino-2-fenylindool) 1:1000 was incubated for 10 min, and quenching of autofluorescence was performed with 0.1% Sudan Black in 70% ethanol for 10 min. Imaging was performed using the Axio Scan Z1 (ZEISS, Oberkochen, Germany) microscope at a magnification of 20×. IF imaging was performed with confocal microscopy on a stimulated emission depletion (STED) microscope at 63× magnification, creating z-stacks of steps of 0.5 µm.

**Table 3:** IHC antibodies

Antibody	Company (cat#)	Clone	Dilution	Fixation buffer
HLA	Abcam (ab7856)	CR3/43	1:1000	4% PFA
PLP	Biorad (MCA8396)	PLC1	1:1500	4% PFA
CD200	Biorad (MCA1960T)	OX-104	1:80 000, 1:400^b^	4% PFA
CD200R	Serotec (MCA2282)	OX-108	1:150^a^, 1:100^a,b^	Acetone
CD47	Serotec (MCA911)	BRIC126	1:30 000, 1:800^b^	4% PFA
Iba1	Wako (019-19741)	Polyclonal	1:100^b^	4% PFA/Acetone
MBP	Millipore (AB980)	Polyclonal	1:100	4% PFA
NF-H	Thermo Fisher (18934-1)	Polyclonal	1:100^a^	4% PFA
SIRPα	Atlas (HPA054437)	Polyclonal	1:400, 1:100^a,b^	4% PFA
NeuN	Sigma (MAB377)	A60	1:500	4% PFA

IF, immunofluorescent; PFA, paraformaldehyde.

^a^Enhanced staining.

^b^IF staining.

Cell types expected to express CD200, CD200R, CD47 and SIRPα were found on the website of the Human Protein Atlas, www.proteinatlas.org, based on integrative omics and single-cell transcriptomics of human tissue,^20,36^ and on the website of Brain RNA-Seq, www.brainrnaseq.org, based on cell type–specific RNA sequencing after immunopanning.^37^

### Immunohistochemistry analysis

Staining analysis was conducted with QuPath (version 0.4.4). Of all sections, the human leukocyte antigens (HLA) and myelin proteolipid protein (PLP) stainings were assessed to confirm absence of pathology in control samples and to annotate regions with GM lesions as well as the adjacent perilesional GM defined as 100 μm expansion from the GM lesions in MS samples. No control donor showed abnormal myelination or microglia activation. For the control donors, a GM region of interest (ROI) was annotated where all layers of the cortex were visible on the neuronal nuclei (NeuN) staining. For MS donors, a NAGM ROI was annotated as far away from GM lesions as possible with intact myelination and where all layers of the cortex were present and visible on the NeuN staining. Sections where the layers could not be distinguished or where the tissue was damaged during staining were excluded.

ROIs were imported into the NeuN images. Based on the NeuN staining, cortical layers were annotated in the ROIs. The first layer of the cortex was recognized by a low neuronal density. The second layer of the cortex was recognized by a high density of small neurons. The third layer of the cortex was recognized by a lower density of neurons and the presence of pyramidal cells. The fourth layer of the cortex was recognized by a high density of small neurons. The fifth layer of the cortex was recognized by a lower density of neuronal cells including large pyramidal cells. The transition from the fifth to the sixth layer of the cortex was recognized by a small rim with a relative increase of the neuronal density. Positive cells were quantified with cell detection and normalized to the size of the ROI.

The annotation of the ROIs including the annotation of the cortical layers was imported into the CD200, CD47 and SIRPα stainings. For CD200, NAWM was additionally annotated. Tissue gaps were eliminated using a thresholder, and for CD200 and CD47, the mean OD was calculated per ROI. For SIRPα, the cells were annotated by nuclei staining-based detection, and within each annotation the number of cells with a DAB intensity exceeding twice the average DAB intensity of that annotation was quantified relative to the area of the annotation. Given that lesions and perilesional GM appeared across diverse cortical layers, and the quantification of CD200, CD47 and SIRPα indicated layer-specific expression, OD or cell density calculations were normalized against NAGM within the same layers. For CD200R, as there were only very few CD200R^+^ cells and these were not homogeneously spread throughout the tissue, and because there was some background staining, the numbers of CD200R^+^ cells were counted manually in lesions and perilesional GM as previously defined as well as in the entire section for NAGM.

### Statistical analysis

All statistics were performed in RStudio Desktop (2023.06.1; RStudio, Boston, MA, USA) using key packages lme4, car, plyr and ggpubr. Missing values were ignored. For IHC, the groups were tested with a quasi-Poisson generalized linear model, statistically correcting for neuronal density when comparing GM lesions with perilesional GM or with NAGM. When more than two conditions were compared, multiple testing correction was performed with false-discovery rate (FDR). For qPCR data, a two-sided *t*-test was performed. Correlations were tested with Pearson’s correlation coefficient. The tests were considered significant if *P* < 0.05.

## Results

### Lower neuronal density in GM lesions

**Figure 1:**
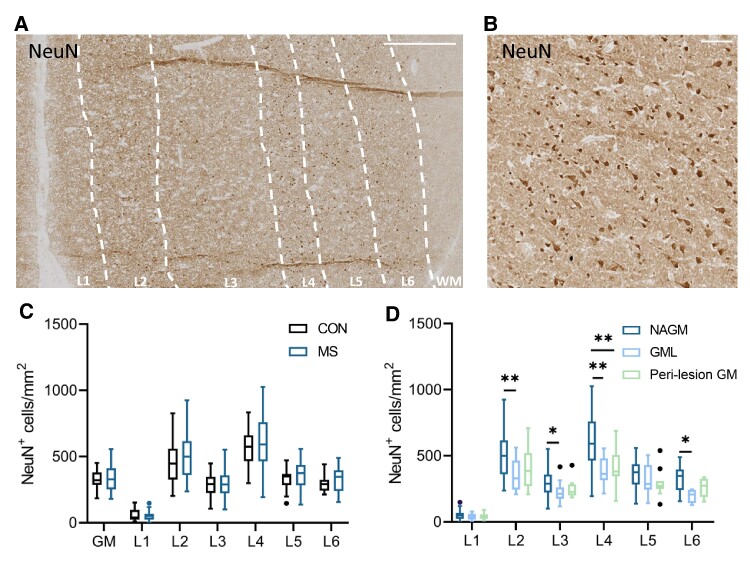
**Neuronal density in the (NA)GM in MS and controls.** (**A**) IHC of NeuN in NAGM in MS, with delineation of the cortical layers (L1–L6) and WM based on neuronal density and neuron shape, scale bar 500 µm. (**B**) Higher magnification of NeuN staining, scale bar 100 µm. (**C**) There was no difference in neuronal density in MS NAGM (*n* = 30) compared with control GM (*n* = 27). (**D**) Neuronal density is lower in GM lesions (*n* = 13) and perilesional GM (*n* = 14) compared with NAGM (*n* = 30) in lesions spanning Layers 2, 3, 4 and 6 of the cortex and in perilesional regions in Layer 4. Box plots indicate the median. * *P* < 0.05, ** *P* < 0.01, *** *P* < 0.001. Significance was tested with a quasi-Poisson generalized linear model, correcting for multiple testing with FDR if comparing multiple groups.

As visualized in Fig. 1A and B, neuronal density was quantified with a NeuN staining, and the layers were separated based on the shape and density of neurons. In MS (*n* = 30) and controls (*n* = 27, the neuronal density in the whole (NA)GM was 327.94 ± 74.36 and 342.23 ± 97.94 neurons/mm^2^, respectively, with the highest neuronal density in Layers 2 and 4. The neuronal density was not different in MS compared with controls in any of the cortical layers or in the whole (NA)GM (Fig. 1C). In GM lesions (*n* = 13) compared with NAGM (*n* = 30), the neuronal density was lower in Layers 2, 3, 4 and 6 of the cortex (Layer 2 NAGM: 492.60 ± 171.57, lesion: 347.30 ± 121.04, *P* = 9.9e−3; Layer 3 NAGM: 293.59 ± 104.28, lesion: 224.11 ± 75.01, *P* = 0.03; Layer 4 NAGM: 599.51 ± 184.66, lesion: 388.93 ± 107.99, *P* = 1.2e−3; and Layer 6 NAGM: 326.02 ± 96.16, lesion: 196.93 ± 52.55, *P* = 0.02). In perilesional GM (*n* = 14) compared with NAGM (*n* = 30), neuronal density was lower in Layer 4 (Layer 4 NAGM: 599.51 ± 184.66, perilesional: 408.50 ± 132.32, *P* = 1.2e−4; Fig. 1D).

### Expression of inhibitory receptor–ligand pairs in the NAGM

**Figure 2:**
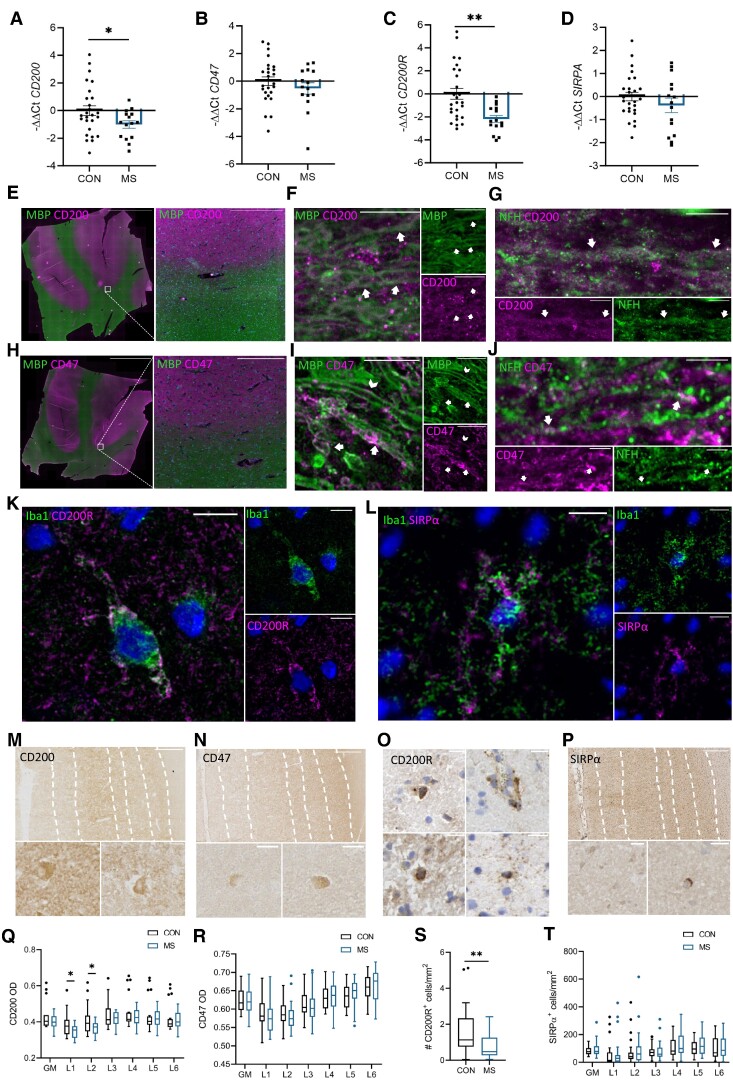
**Gene and protein expression of CD200, CD47, CD200R and SIRPα in NAGM.** Gene expression in NAGM of MS compared with GM of controls as measured with qRT-PCR (MS *n* = 15, CON *n* = 26) was (**A**) lower for *CD200*, (**B**) comparable for *CD47*, (**C**) lower for *CD200R* and (**D**) comparable for *SIRPA*. (**E**) Immunofluorescent staining of CD200 and MBP shows that CD200 expression was abundant in NAGM and low in NAWM. Scale bar on the overview image is 1 cm and on the zoomed image it is 250 µm. (**F**) CD200 expression co-localized with MBP^+^ myelin as indicated with arrows; scale bar is set at 10 µm. (**G**) CD200 expression co-localized with NFH^+^ axons as indicated with arrows; scale bar is set at 5 µm. (**H**) CD47 expression was, like CD200, abundant in NAGM and lower in NAWM. Scale bar on the overview image is 1 cm and on the zoomed image is 250 µm. (**I**) CD47 strongly co-localized with some MBP^+^ myelin, as indicated with the arrows, and was sometimes localized adjacent to the myelin, on the outer layer, as indicated with the arrowhead. Scale bar is set at 10 µm. (**J**) Occasionally, CD47 co-localized with NFH^+^ axons, scale bar is set at 5 µm. (**K**) CD200R was expressed by Iba1^+^ microglia; scale bar is set at 10 µm. (**L**) SIRPα was expressed by Iba1^+^ microglia; scale bar is set at 10 µm. DAB stainings showed (**M**) mainly extracellular deposition and sporadic expression on neurons for CD200, (**N**) mainly extracellular deposition and sporadic expression on neurons for CD47, (**O**) sporadic positive staining of cells for CD200R, and (**P**) light positive neurons and some darker stained round cells for SIRPα. Scale bars are set at 20 µm. In MS NAGM compared with control GM, there was a (**Q**) lower CD200 OD in cortical Layers 1 and 2 (MS *n* = 19, CON *n* = 19), (**R**) comparable CD47 OD (MS *n* = 31, CON *n* = 28), (**S**) lower number of CD200R^+^ cells/mm^2^ (MS *n* = 24, CON *n* = 21), and (**T**) comparable number of SIRPα^+^ cells/mm^2^ (MS *n* = 31, CON *n* = 25). Box plots indicate the median. * *P* < 0.05, ** *P* < 0.01, *** *P* < 0.001. Significance was tested with a quasi-Poisson generalized linear model.

Expression of CD200, CD200R, CD47 and SIRPα in (NA)GM was quantified with qRT-PCR and IHC. The expression of CD200, CD200R, CD47 and SIRPα was not correlated to the neuronal density (data not shown). As visualized in Fig. 2A–D, RNA expression of both *CD200* and *CD200R* was lower in MS NAGM (*n* = 15) compared with control GM (*n* = 26) (*CD200*: −ΔΔCT CON: 0.00 ± 1.82, −ΔΔCT MS: −1.00 ± 1.05, *P* = 0.03; *CD200R*: −ΔΔCT CON: 0.00 ± 2.40, −ΔΔCT MS: −2.19 ± 1.18, *P* = 3.6e−4). No difference in RNA expression was observed for *CD47* or *SIRPA*.

According to the Human Protein Atlas (v.23.0.proteinatlas.org)^20,36^ and the Brain RNA-Seq database,^37^ CD200 and CD47 are highly expressed by neurons and to a lower extent by oligodendrocytes. CD47 is furthermore also expressed by astrocytes.^20,36,37^ CD200R is very lowly expressed by microglia, T and B cells, neurons, astrocytes and oligodendrocytes.^20,36,37^ SIRPα is expressed by microglia and barely by T and B cells, neurons, astrocytes and oligodendrocytes.^20,36,37^ With IF, we show that CD200 and CD47 both were abundantly expressed in NAGM and to a lesser extent in NAWM in a similar pattern. The staining was highly intense and so diffuse that cellular localization was difficult to interpret. Therefore, we stained NAWM in the pyramid tract, where lower expression of CD200 and CD47 enables visualization of cellular localization. We found faint but distinct co-localization of CD200 with myelin and axons. Myelin also frequently stained strongly positive for CD47, which might originate from myelin-forming oligodendrocytes. Infrequently, CD47^+^ axonal fragments were found (Fig. 2E–J). CD200R as well as SIRPα were co-localized with the microglia/macrophage marker Iba1 (Fig. 2K–L). CD200R^+^Iba1^−^ cells were rare, while SIRPα^+^Iba1^−^ cells were observed more frequently.

Representative DAB immunohistochemical stainings for CD200, CD47, CD200R and SIRPα are visualized in Fig. 2M–P. The majority of CD200 was found extracellularly, and occasionally CD200^+^ neuronal cell bodies could be identified. CD200 was visually higher in the (NA)GM than in the adjacent WM. In the (NA)GM, the lowest amount of CD200 was found in Layers 1 and 2 of the cortex, which in MS, but not in controls, was significantly lower compared with Layers 3, 4, 5 and 6 (MS L1: OD 0.35 ± 0.04 and L2: OD 0.37 ± 0.04 versus L3: OD 0.42 ± 0.04, *P* < 0.0001, *P* = 5.0e−4, respectively, L4: OD 0.43 ± 0.05, *P* < 0.0001, *P* < 0.0001, respectively, L5: OD 0.42 ± 0.05, *P* < 0.0001, *P* = 2.0e−4, respectively, L6: OD 0.41 ± 0.05, *P* < 0.0001, *P* = 2.5e−3, respectively). In MS (*n* = 19) compared with controls (*n* = 19), no difference in OD of CD200 was found when the whole GM was compared; however, there was a significantly lower OD of CD200 in Layers 1 and 2 (Layer 1 controls: 0.39 ± 0.07, MS: 0.35 ± 0.04, *P* = 0.03; Layer 2 controls: 0.41 ± 0.08, MS: 0.37 ± 0.04, *P* = 0.04). In the other layers, no difference was found between MS and controls (Fig. 2Q).

CD47 was found abundantly expressed extracellularly throughout the (NA)GM and sporadically on neuronal cell bodies, like CD200. CD47 expression visually was lower in WM than in the (NA)GM. OD of CD47 was similar in Layers 1 and 2 and was significantly higher per consecutive layer except from Layers 4 to 5 in both control GM as well as MS NAGM (CON L1: 0.59 ± 0.04, L2: 0.59 ± 0.03, L1 versus L2 *P* = *n.s.*, L3: 0.61 ± 0.03, L2 versus L3 *P* = 0.03, L4: 0.63 ± 0.03, L3 versus L4 *P* = 0.03, L5: 0.65 ± 0.03, L4 versus L5 *P* = *n.s*., L6: 0.66 ± 0.04, L5 versus L6 *P* = 0.01; MS L1: 0.58 ± 0.04, L2: 0.58 ± 0.04, L1 versus L2 *P* = *n.s.*, L3: 0.61 ± 0.04, L2 versus L3 *P* = 9.9E^−3^, L4: 0.63 ± 0.04, L3 versus L4 *P* = 0.02, L5: 0.64 ± 0.04, L4 versus L5 *P* = *n.s*., L6: 0.66 ± 0.04, L5 versus L6 *P* = *n.s.*). No difference was found in CD47 OD in MS NAGM (*n* = 31) compared with control GM (*n* = 28) (Fig. 2R).

For CD200R, the number of positive cells, likely microglia and macrophages, was quantified in the whole (NA)GM, with no distinction between layers. CD200R^+^ cells were only found sporadically. The majority of CD200R^+^ cells were found perivascular, but sporadically CD200R^+^ cells were found in the parenchyma. There was a small but significant lower number of CD200R^+^ cells/mm^2^ in NAGM (*n* = 24) compared with control GM (*n* = 21) (control GM: 1.60 ± 1.44, MS NAGM: 0.75 ± 0.67, *P* = 9.2e−3, Fig. 2S).

SIRPα, similarly to CD47, gradually increased from Layers 1 to 6 in both MS and controls, similarly to CD47, but this was not significant in either controls or MS. Strong SIRPα expression was found on round parenchymal cells, which are likely microglia and lymphocytes, and low SIRPα expression was found on neurons. No difference was found in the (NA)GM or in the layers in MS (*n* = 31) compared with controls (*n* = 25) (Fig. 2T).

### Lower expression of CD200–CD200R and CD47–SIRPα in GM lesions and perilesional GM

**Figure 3:**
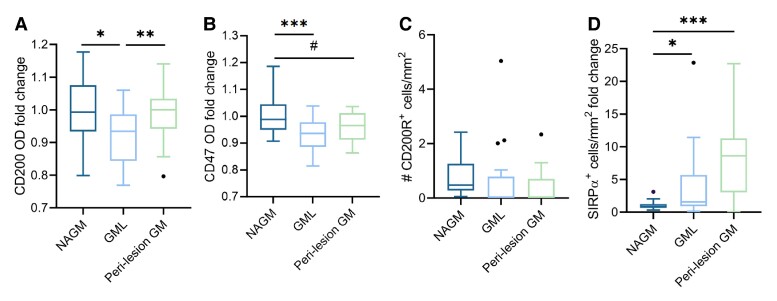
**Distribution of CD200, CD47, CD200R and SIRPα in GM lesions and perilesional GM. (A)** CD200 OD was lower in GM lesions (*n* = 16) compared with NAGM and perilesional GM (*n* = 19). (**B**) CD47 OD was lower in GM lesions (*n* = 24) compared with NAGM (*n* = 24). (**C**) There was no difference in number of CD200R^+^ cells/mm^2^ in GM lesions (*n* = 24) or perilesional GM (*n* = 18) compared with NAGM (*n* = 18). (**D**) In GM lesions (*n* = 17) and perilesional GM (*n* = 15), there was an increase in the number of SIRPα^+^ cells/mm^2^ compared with the NAGM (*n* = 31). Box plots indicate the median. * *P* < 0.05, ** *P* < 0.01, *** *P* < 0.001, # *P* = 0.05. Significance was tested with a quasi-Poisson generalized linear model, correcting for neuronal density and for multiple testing with FDR.

In GM lesions (*n* = 16), CD200 was lower compared with NAGM (*n* = 19) (fold change OD: 0.92 ± 0.08, *P* = 0.02, Fig. 3A). CD200 expression in perilesional GM (*n* = 16) was like in NAGM (*n* = 19). CD47 was lower in GM lesions (*n* = 24) (fold change GM lesions compared with NAGM: 0.93 ± 0.01, *P* = 2.0e−4) and near-significantly lower in perilesional GM (*n* = 24) compared with NAGM (*n* = 24) (fold change perilesional GM compared with NAGM: 0.96 ± 0.01, *P* = 0.05, Fig. 3B). There was no difference in the number of CD200R^+^ cells/mm^2^ in NAGM (*n* = 24), GM lesions (*n* = 18), or perilesional GM (*n* = 18) (Fig. 3C). SIRPα expression was increased in GM lesions (*n* = 17) compared with NAGM (*n* = 31) (fold change GM lesions compared with NAGM: 5.96 ± 9.55, *P* = 0.01) and further increased in perilesional region (*n* = 15) (fold change perilesional compared with NAGM: 7.65 ± 5.92, *P* = 6.1e−4, Fig. 3D).

### Neuronal density and CD200 expression in NAGM correlate with MS donor pathology

**Figure 4:**
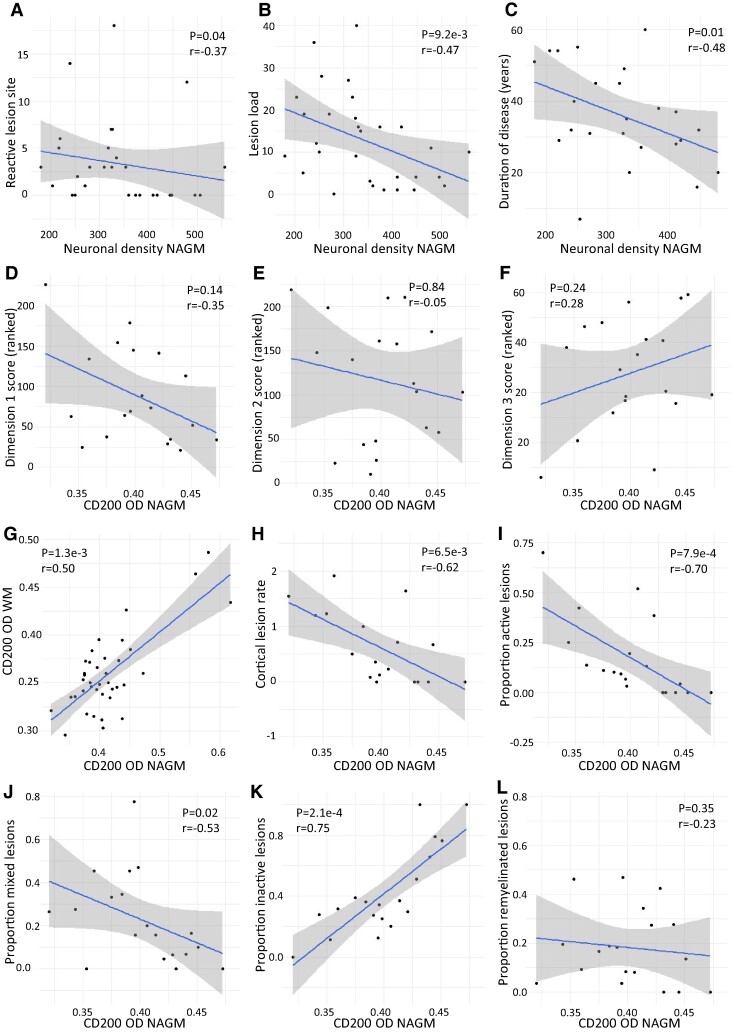
**Neuronal density and CD200 OD of NAGM in MS correlate with pathology.** Neuronal density was negatively correlated to (**A**) the reactive site load, (**B**) the lesion load and (**C**) the disease duration (*n* = 30). CD200 OD in NAGM showed (**D**) a trend towards a negative correlation with demyelination and immune cell activity (Dimension 1), (**E**) no correlation with microglia (re)activity and possibly lesion initiation (Dimension 2) and (**F**) a trend towards a positive correlation with loss of lesion activity and scar formation (Dimension 3). (**G**) The OD of CD200 in WM was positively correlated to the OD of CD200 in GM (*n* = 19). CD200 OD was negatively correlated to (**H**) the cortical lesion rate, (**H**) the ratio of leukocortical lesions, (**I**) the proportion of active lesions and (**J**) the proportion of mixed active/inactive lesions (*n* = 19). CD200 OD was positively correlated to (**K**) the proportion of inactive lesions and was not correlated to (**L**) the proportion of remyelinated lesions (*n* = 19). Significance was tested with a Spearman’s correlation.

In NAGM, neuronal density negatively correlated to the reactive site load (*P* = 0.04, *r* = −0.37, Fig. 4A) and to the lesion load (*n* = 30) (*P* = 9.2e−3, *r* = −0.47, Fig. 4B). The neuronal density of NAGM negatively correlated with the disease duration (*P* = 0.01, *r* = −0.48, Fig. 4C), also after correction for age (*P* = 0.05). Recently, exploratory factor analysis identified three pathologically and clinically relevant dimensions of disease progression.^33^ Although the correlations were not statistically significant, there was a noticeable trend towards a negative correlation with demyelination and immune cell activity (Dimension 1), no correlation with microglia (re)activity and possibly lesion initiation (Dimension 2) and a trend towards a positive correlation with loss of lesion activity and scar formation (Dimension 3) (Fig. 4D–F). These trends could indicate a negative association between CD200 and ongoing microglia activation and demyelination. The OD of CD200 in NAGM (*n* = 19) was correlated to the OD of CD200 in NAWM (*P* = 1.3e−3, *r* = 0.50, Fig. 4G). CD200 NAGM OD negatively correlated with the cortical lesion rate (*P* = 6.5e−3, *r* = −0.62, Fig. 4H). CD200 NAGM OD furthermore correlated negatively with the proportion of active lesions (*P* = 7.9e−4, *r* = −0.70, Fig. 4I), mixed active/inactive lesions (*P* = 0.02, *r* = −0.53, Fig. 4J), was correlated positively with the proportion of inactive lesions (*P* = 2.1e−4, *r* = 0.75, Fig. 4K) and was not correlated to the proportion of remyelinated lesions (*P* = 0.35, *r* = −0.23, Fig. 4L). The OD of CD47, the number of SIRPα^+^ cells/mm^2^ and the number of CD200R^+^ cells/mm^2^ were not correlated to any pathological characteristics. The OD of CD200 and CD47 and the number of SIRPA^+^ cells or CD200R^+^ cells per mm^2^ were not correlated to disease duration.

## Discussion

GM lesions have a pathogenesis that differs from the pathogenesis of WM lesions. In contrast to WM lesions, there are only few T cells that infiltrate the parenchyma. Also, there are fewer microglia, and these microglia tend to be less pro-inflammatory.^6,9,38,39^ Neuronal cell bodies in GM expressing CD200 and CD47 may be in part responsible for keeping the microglia in the cortical GM in a more homeostatic state when compared with WM. The binding of CD200 and CD47 to their respective microglial receptors has immunosuppressive effects, maintaining microglia in a ramified, antiphagocytic state.^19,26^ Here, we investigate the RNA and protein expression of the immune-inhibitory ligands C200 and CD47 and of their respective receptors, CD200R and SIRPα, in NAGM, GM lesions and perilesional GM, and relate these to pathological features of the donors, with the aim of unveiling mechanisms involved in microglia activation and MS-related pathology.

Although the neuronal density between MS and control (NA)GM was comparable, GM lesions and perilesional GM exhibited lower neuronal density, which is similar to what was previously found.^12,13^ Decreased perilesional neuronal density indicates that neuronal damage extends beyond the demyelinated region of the lesion. As CD200, CD200R, CD47 and SIRPα are all expressed by neurons to some extent, the altered expression of these proteins in lesions and perilesional GM might be influenced by the lower neuronal density. Therefore, we corrected for the neuronal density in the statistical model. This does not, however, correct for loss of neuronal processes and synapses, which may influence the data. The neuronal density negatively correlated with both the lesion load and the duration of disease, also after correction for age, which may be due to dying back of neurons.

From RNA sequencing studies, it is known that CD200 and CD47 are expressed by neurons and, more so for CD47, by oligodendrocytes.^20,36,37^ CD47 is furthermore also expressed by astrocytes. Our immunohistochemical stainings show a pattern for CD200 and CD47 that is highly intense in the (NA)GM and less intense in the (NA)WM, and we additionally show that both, CD200 and CD47, are co-expressed with myelin and axons. Additionally, we show that CD47 is localized adjacent to myelin. CD200R mRNA and protein are lowly expressed,^20,27,36,37^ mainly by microglia, whereas microglia robustly express SIRPα.^20,36,37^ Both receptors are also expressed by T and B cells, neurons, astrocytes and oligodendrocytes, indicating their diverse effects.^20,36,37^ Here, with IHC, we validated that CD200R and SIRPα are indeed expressed by microglia. The low mRNA expression of CD200R indicates either a very low basic protein presence or expression by only a small fraction of cells. Indeed, we found that only very few microglia expressed CD200R, and it is possible that we cannot accurately quantify all CD200R^+^ microglia with IHC. SIRPα, in line with its more abundant mRNA expression, was found on a larger number of microglia.

The CD200R cytoplasmic tail bears three tyrosine residues, which, upon CD200–CD200R interaction, undergo phosphorylation, initiating downstream inhibitory protein responses via Dok1 and Dok2. This leads to the downregulation of pro-inflammatory cytokines, including TNF, IFN-γ and IL-1, alongside upregulation of anti-inflammatory cytokines, such as IL-10 and TGF-β, consequently inhibiting phagocytosis.^40^ The upstream mechanisms of CD200 regulation are not yet fully understood and need further investigation. In Alzheimer’s disease, a loss of CD200 expression is hypothesized to contribute to chronic inflammation.^41^ Notably, we found that CD200 and CD200R gene expression was lower in MS NAGM compared with control GM, and CD200 OD was lower in cortical Layers 1 and 2. Similar to what we previously found in WM lesions,^26^ CD200 OD was lower in GM lesions compared with NAGM, even when correcting for neuronal density, and not in perilesional GM compared with NAGM. Interestingly, here we show that there was a negative correlation between the OD of CD200 in NAGM and the cortical lesion rate. The lower expression of CD200 in NAGM and GM lesions may indicate a decrease in the control of pro-inflammatory cytokines and phagocytosis; therefore, lower CD200 may lead to the cortex being more vulnerable to the initiation of lesion formation. Previously, we have shown that the presence of GM lesions was associated with more extensive MS pathology in WM.^8^ We found that CD200 OD in NAGM correlated with the CD200 OD in NAWM, which could point to a donor-related lower CD200 expression relating to both GM- and WM-related pathology. Despite lacking statistical significance, the directionality of the correlation between CD200 in NAGM and three recently identified dimensions of disease progression^33^ indicates that CD200 expression is negatively associated with ongoing microglia activity and demyelination. In line with this, here we show that lower CD200 OD in GM is associated with more actively demyelinating MS pathology in WM: CD200 OD of NAGM is negatively correlated with the proportion of active and mixed active/inactive, and positively correlated with the proportion of inactive lesions. It remains elusive if CD200 expression is associated with GM lesion activity, as inflammatory cortical demyelination is mainly associated with lymphocyte infiltration in the meninges,^42^ which have for the majority been removed during autopsy.

Previously, it has been shown that in EAE, a mouse model for autoimmune inflammatory diseases of the CNS, subcutaneous administration of a dimeric CD200R fusion protein (CD200R-Fc) that interacts with CD200R1 as agonist, can prevent demyelination and reduce symptom burden.^31^ In a rat model for chronic constriction injury of the sciatic nerve, intrathecal CD200R-Fc application attenuated activation of microglial cells, decreased pro-inflammatory cytokine messenger RNA levels and increased anti-inflammatory cytokine messenger RNA levels.^43^ In human renal cells *in vitro*, CD200R-Fc attenuated the inflammatory response to lipopolysaccharide.^44^ Rat monoclonal antibodies agonistic for human CD200R as well as CD200R-Fc administered *in vitro* to mouse peritoneal cells, human peripheral blood cells, human dendritic cells and a human monocyte cell line activated with IFN-y inhibit pro-inflammatory cytokine production, especially after further cross-linking of the agonists.^45^ Polyethylene glycol-modified DNA aptamer is also a CD200R1 agonist and can suppress the induction of cytotoxic T lymphocytes in human and murine allogeneic-mixed lymphocyte cultures.^46^ We hypothesize that elevated expression of CD200 may lead to resolution of microglia activation, thereby potentially inhibiting smouldering lesion activity. The lack of correlation between CD200 OD of NAGM and the proportion of remyelinated lesions indicates that CD200 upregulation will likely not inhibit microglia activity supporting remyelination. Additionally, as oligodendrocytes express CD200, myelin may also express CD200, which could directly inhibit phagocytosis of myelin. Lastly, as microglia activation precedes MS lesion formation,^47^ and CD200 suppresses activation of microglia, activation of the CD200–CD200R pathway could be an interesting therapeutic target to decrease overall microglia activation and may limit the formation of new lesions. There are also facts that question this hypothesis, in particular the very low expression of CD200R by microglia and recent data showing that IFN-α can rewire CD200R signalling to become pro-inflammatory.^48^ The biological role of CD200R therefore needs further investigation.

SIRPα contains intracellular immunoreceptor tyrosine-based inhibitory motifs, which, upon phosphorylation due to CD47 binding, recruit phosphatases like SH2-domain-containing protein tyrosine phosphatase (SHP-2) leading to inhibition of phagocytosis through deactivation of the motor protein myosin IIA.^23,24^ Myosin significantly influences phagocytosis by modulating actin filaments.^49,50^ Recently, SIRPα-Fc and less potently CD47-Fc were shown to have anti-inflammatory effects.^51^  *In vitro* administration of SIRPα-Fc and high concentrations of CD47-Fc to a macrophage cell line suppressed inflammatory cytokine secretion in a cross-linking dependent manner, similar to the effect observed with CD200R-Fc. *In vivo*, intraperitoneally administered SIRPα-Fc showed antichemotactic effects on neutrophils and monocytes. In an experimental arthritis model, SIRPα-Fc reduced arthritis severity by ameliorating joint inflammation via inhibition of neutrophil and monocyte infiltration.^51^ In line with this, *in vivo* administration of Miap410, a CD47-blocking antibody, in EAE impaired resolution of CNS inflammation.^52^ We here show that CD47 is highly expressed in the (NA)GM. In NAWM of the pyramid tract, we additionally show that CD47 can co-localize with myelin or be found adjacent to myelin. CD47 gene expression and OD as well as SIRPα gene expression and positive cell density showed no significant differences between MS and control donors. However, CD47 OD was significantly lower in GM lesions compared with NAGM, and near-significantly lower in perilesional GM, which together suggests a role in lesion progression. By contrast, there was an increase in the number of SIRPα^+^ cells/mm^2^ in GM lesions as well as in perilesional GM, possibly reflecting a neuroprotective response or an increase in microglia in these lesions. Taken together, CD47 is not likely associated with the initiation of GM lesion formation; however, it may be involved in the expansion of GM lesions. Therefore, possibly CD47 and/or SIRPα may represent interesting therapeutic targets to reduce the expansion of GM lesions.

## Conclusion

We show that downregulated CD200 expression in MS NAGM is associated with increased cortical lesion rate and more severe WM pathology of the donors. Meanwhile, CD47 is downregulated in GM lesions and in perilesional GM, implicating its involvement in lesion progression. These findings provide more insights into the mechanisms behind GM lesion formation and progression and underscore the putative therapeutic potential of checkpoint molecules in mitigating pathological progression in both WM and GM.

## Supplementary Material

fcae264_Supplementary_Data

## Data Availability

All gene expression data as well as IHC data are available upon reasonable request. The script for data analysis is provided as Supplementary material.

## References

[1] Lassmann H, Van Horssen J, Mahad D. Progressive multiple sclerosis: Pathology and pathogenesis. Nat Rev Neurol. 2012;8(11):647–656. 23007702 10.1038/nrneurol.2012.168

[2] Dendrou CA, Fugger L, Friese MA. Immunopathology of multiple sclerosis. Nat Rev Immunol. 2015;15(9):545–558. 26250739 10.1038/nri3871

[3] Lassmann H . Pathogenic mechanisms associated with different clinical courses of multiple sclerosis. Front Immunol. 2019;10(Jan):3116. 30687321 10.3389/fimmu.2018.03116 PMC6335289

[4] van der Poel M, Ulas T, Mizee MR, et al Transcriptional profiling of human microglia reveals grey–white matter heterogeneity and multiple sclerosis-associated changes. Nat Commun. 2019;10(1):1139. 30867424 10.1038/s41467-019-08976-7 PMC6416318

[5] van Olst L, Rodriguez-Mogeda C, Picon C, et al Meningeal inflammation in multiple sclerosis induces phenotypic changes in cortical microglia that differentially associate with neurodegeneration. Acta Neuropathol. 2021;141(6):881–899. 33779783 10.1007/s00401-021-02293-4 PMC8113309

[6] Bö L, Geurts JJG, Mörk SJ, Van Der Valk P. Grey matter pathology in multiple sclerosis. Acta Neurol Scand. 2006;113(Suppl 183):48–50. 10.1111/j.1600-0404.2006.00615.x 16637929

[7] Albert M, Antel J, Brück W, Stadelmann C. Extensive cortical remyelination in patients with chronic multiple sclerosis. Brain Pathol. 2007;17(2):129–138. 17388943 10.1111/j.1750-3639.2006.00043.x PMC8095564

[8] Luchetti S, Fransen NL, van Eden CG, Ramaglia V, Mason M, Huitinga I. Progressive multiple sclerosis patients show substantial lesion activity that correlates with clinical disease severity and sex: A retrospective autopsy cohort analysis. Acta Neuropathol. 2018;135(4):511–528. 29441412 10.1007/s00401-018-1818-y PMC5978927

[9] Stadelmann C, Albert M, Wegner C, Brück W. Cortical pathology in multiple sclerosis. Curr Opin Neurol. 2008;21(3):229–234. 18451703 10.1097/01.wco.0000318863.65635.9a

[10] Kutzelnigg A, Lucchinetti CF, Stadelmann C, et al Cortical demyelination and diffuse white matter injury in multiple sclerosis. Brain. 2005;128(11):2705–2712. 16230320 10.1093/brain/awh641

[11] Bø L, Vedeler CA, Nyland HI, Trapp BD, Mørk SJ. Subpial demyelination in the cerebral cortex of multiple sclerosis patients. J Neuropathol Exp Neurol. 2003;62(7):723–732. 12901699 10.1093/jnen/62.7.723

[12] Peterson JW, Bö L, Mörk S, Chang A, Trapp BD. Transected neurites, apoptotic neurons, and reduced inflammation in cortical multiple sclerosis lesions. Ann Neurol. 2001;50(3):389–400. 11558796 10.1002/ana.1123

[13] Klaver R, Popescu V, Voorn P, et al Neuronal and axonal loss in normal-appearing gray matter and subpial lesions in multiple sclerosis. J Neuropathol Exp Neurol. 2015;74(5):453–458. 25853695 10.1097/NEN.0000000000000189

[14] Calabrese M, Agosta F, Rinaldi F, et al Cortical lesions and atrophy associated with cognitive impairment in relapsing-remitting multiple sclerosis. Arch Neurol. 2009;66(9):1144–1150. 19752305 10.1001/archneurol.2009.174

[15] Tsouki F, Williams A. Multifaceted involvement of microglia in gray matter pathology in multiple sclerosis. Stem Cells. 2021;39(8):993–1007. 33754376 10.1002/stem.3374

[16] Colonna M, Butovsky O. Microglia function in the central nervous system during health and neurodegeneration. Annu Rev Immunol. 2017;35:441–468. 28226226 10.1146/annurev-immunol-051116-052358 PMC8167938

[17] Szepesi Z, Manouchehrian O, Bachiller S, Deierborg T. Bidirectional microglia–neuron communication in health and disease. Front Cell Neurosci. 2018;12(Sep):323. 30319362 10.3389/fncel.2018.00323 PMC6170615

[18] Li Q, Barres B. Microglia and macrophages in brain homeostasis and disease. Nat Rev Immunol. 2018;18:225–242. 29151590 10.1038/nri.2017.125

[19] Biber K, Neumann H, Inoue K, Boddeke HWGM. Neuronal “on” and “off” signals control microglia. Trends Neurosci. 2007;30(11):596–602. 17950926 10.1016/j.tins.2007.08.007

[20] Karlsson M, Zhang C, Méar L, et al A single–cell type transcriptomics map of human tissues. Sci Adv. 2021;7(31):eabh2169. 34321199 10.1126/sciadv.abh2169 PMC8318366

[21] Lehrman EK, Wilton DK, Litvina EY, et al CD47 protects synapses from excess microglia-mediated pruning during development. Neuron. 2018;100(1):120–134. 30308165 10.1016/j.neuron.2018.09.017 PMC6314207

[22] Hoek RH, Ruuls SR, Murphy CA, et al Down-regulation of the macrophage lineage through interaction with OX2 (CD200). Science. 2000;290(5497):1768–1771. 11099416 10.1126/science.290.5497.1768

[23] Barclay AN, Van Den Berg TK. The interaction between signal regulatory protein alpha (SIRPα) and CD47: Structure, function, and therapeutic target. Annu Rev Immunol. 2014;32(October):25–50. 24215318 10.1146/annurev-immunol-032713-120142

[24] Logtenberg MEW, Scheeren FA, Schumacher TN. The CD47-SIRPα immune checkpoint. Immunity. 2020;52(5):742–752. 32433947 10.1016/j.immuni.2020.04.011 PMC7340539

[25] Scheepstra KWF, Mizee MR, van Scheppingen J, et al Microglia transcriptional profiling in major depressive disorder shows inhibition of cortical gray matter microglia. Biol Psychiatry. 2023;94(8):619–629. 37121366 10.1016/j.biopsych.2023.04.020

[26] Koning N, Bö L, Hoek RM, Huitinga I. Downregulation of macrophage inhibitory molecules in multiple sclerosis lesions. Ann Neurol. 2007;62(5):504–514. 17879969 10.1002/ana.21220

[27] Koning N, Swaab DF, Hoek RM, Huitinga I. Distribution of the immune inhibitory molecules CD200 and CD200R in the normal central nervous system and multiple sclerosis lesions suggests neuron-glia and glia-glia interactions. J Neuropathol Exp Neurol. 2009;68(2):159–167. 19151626 10.1097/NEN.0b013e3181964113

[28] Valente T, Serratosa J, Perpiñá U, Saura J, Solà C. Alterations in CD200-CD200R1 system during EAE already manifest at presymptomatic stages. Front Cell Neurosci. 2017;11(May):129. 28522962 10.3389/fncel.2017.00129 PMC5415594

[29] Meuth SG, Simon OJ, Grimm A, et al CNS inflammation and neuronal degeneration is aggravated by impaired CD200-CD200R-mediated macrophage silencing. J Neuroimmunol. 2008;194(1–2):62–69. 18164423 10.1016/j.jneuroim.2007.11.013

[30] Chitnis T, Imitola J, Wang Y, et al Elevated neuronal expression of CD200 protects Wlds mice from inflammation-mediated neurodegeneration. Am J Pathol. 2007;170(5):1695–1712. 17456775 10.2353/ajpath.2007.060677 PMC1854964

[31] Liu Y, Bando Y, Vargas-Lowy D, et al CD200R1 agonist attenuates mechanisms of chronic disease in a murine model of multiple sclerosis. J Neurosci. 2010;30(6):2025–2038. 20147531 10.1523/JNEUROSCI.4272-09.2010 PMC2837938

[32] Han MH, Lundgren DH, Jaiswa S, et al Janus-like opposing roles of CD47 in autoimmune brain inflammation in humans and mice. J Exp Med. 2012;209(7):1325–1334. 22734047 10.1084/jem.20101974 PMC3405500

[33] de Boer A, van den Bosch AMR, Mekkes NJ, et al Disentangling the heterogeneity of multiple sclerosis through identification of independent neuropathological dimensions. Acta Neuropathol. 2024;147:90. 38771530 10.1007/s00401-024-02742-w PMC11108935

[34] Mekkes NJ, Groot M, Hoekstra E, et al Identification of clinical disease trajectories in neurodegenerative disorders with natural language processing. Nat Med. 2024;30(4):1143–1153. 38472295 10.1038/s41591-024-02843-9 PMC11031398

[35] Harroud A, Stridh P, McCauley JL, et al Locus for severity implicates CNS resilience in progression of multiple sclerosis. Nature. 2023;619:323–331. 37380766 10.1038/s41586-023-06250-x PMC10602210

[36] Sjöstedt E, Zhong W, Fagerberg L, et al An atlas of the protein-coding genes in the human, pig, and mouse brain. Science (80-). 2020;367:eaay5947. 10.1126/science.aay5947 32139519

[37] Zhang Y, Sloan SA, Clarke LE, et al Purification and characterization of progenitor and mature human astrocytes reveals transcriptional and functional differences with mouse. Neuron. 2016;89(1):37–53. 26687838 10.1016/j.neuron.2015.11.013 PMC4707064

[38] De Stefano N, Matthews PM, Filippi M, et al Evidence of early cortical atrophy in MS: Relevance to white matter changes and disability. Neurology. 2003;60(7):1157–1162. 12682324 10.1212/01.wnl.0000055926.69643.03

[39] Prins M, Schul E, Geurts J, van der Valk P, Drukarch B, van Dam AM. Pathological differences between white and grey matter multiple sclerosis lesions. Ann N Y Acad Sci. 2015;1351(1):99–113. 26200258 10.1111/nyas.12841

[40] Manich G, Recasens M, Valente T, Almolda B, González B, Castellano B. Role of the CD200-CD200R axis during homeostasis and neuroinflammation. Neuroscience. 2019;405:118–136. 30367946 10.1016/j.neuroscience.2018.10.030

[41] Walker DG, Dalsing-hernandez JE, Campbell NA, Lue L. Decreased expression of CD200 and CD200 receptor in Alzheimer’s disease: A potential mechanism leading to chronic inflammation. Exp Neurol. 2009;215(1):5–19. 18938162 10.1016/j.expneurol.2008.09.003 PMC2765462

[42] Lucchinetti CF, Popescu BFG, Bunyan RF, et al Inflammatory cortical demyelination in early multiple sclerosis. N Engl J Med. 2011;365(23):2188–2197. 22150037 10.1056/NEJMoa1100648 PMC3282172

[43] Hernangómez M, Klusáková I, Joukal M, Hradilová-Svíženská I, Guaza C, Dubovỳ P. CD200R1 agonist attenuates glial activation, inflammatory reactions, and hypersensitivity immediately after its intrathecal application in a rat neuropathic pain model. J Neuroinflammation. 2016;13(1):43. 26891688 10.1186/s12974-016-0508-8 PMC4759712

[44] Ding Y, Yang H, Xiang W, He X, Liao W, Yi Z. CD200R1 agonist attenuates LPS-induced inflammatory response in human renal proximal tubular epithelial cells by regulating TLR4-MyD88-TAK1-mediated NF-κB and MAPK pathway. Biochem Biophys Res Commun. 2015;460(2):287–294. 25791482 10.1016/j.bbrc.2015.03.026

[45] Jenmalm MC, Cherwinski H, Bowman EP, Phillips JH, Sedgwick JD. Regulation of myeloid cell function through the CD200 receptor. J Immunol. 2006;176(1):191–199. 16365410 10.4049/jimmunol.176.1.191

[46] Prodeus A, Sparkes A, Fischer NW, et al A synthetic cross-species CD200R1 agonist suppresses inflammatory immune responses in vivo. Mol Ther Nucleic Acids. 2018;12(Sep):350–358. 30195773 10.1016/j.omtn.2018.05.023 PMC6037911

[47] van den Bosch AMR, van der Poel M, Fransen NL, et al Profiling of microglia nodules in multiple sclerosis reveals propensity for lesion formation. Nat Commun. 2024;15(1):1667. 38396116 10.1038/s41467-024-46068-3 PMC10891081

[48] van der Vlist M, Ramos MIP, van den Hoogen LL, et al Signaling by the inhibitory receptor CD200R is rewired by type I interferon. Sci Signal. 2021;14(704):eabb4324. 34637328 10.1126/scisignal.abb4324

[49] Stendahl OI, Hartwig JH, Brotschi EA, Stossel TP. Distribution of actin-binding protein and myosin in macrophages during spreading and phagocytosis. J Cell Biol. 1980;84(2):215–224. 6991506 10.1083/jcb.84.2.215 PMC2110553

[50] Tsai RK, Discher DE. Inhibition of “self” engulfment through deactivation of myosin-II at the phagocytic synapse between human cells. J Cell Biol. 2008;180(5):989–1003. 18332220 10.1083/jcb.200708043 PMC2265407

[51] Xie MM, Dai B, Hackney JA, et al An agonistic anti-signal regulatory protein α antibody for chronic inflammatory diseases. Cell Reports Med. 2023;4(8):101130. 10.1016/j.xcrm.2023.101130 PMC10439247 37490914

[52] Wang H, Newton G, Wu L, et al CD47 antibody blockade suppresses microglia-dependent phagocytosis and monocyte transition to macrophages, impairing recovery in EAE. JCI Insight. 2021;6(21):e148719. 34591795 10.1172/jci.insight.148719 PMC8663579

