# Supplementary material for: Cortical CD200–CD200R and CD47–SIRPα expression is associated with multiple sclerosis pathology
Source: Brain Commun. 2024 Aug 7;6(4):fcae264. doi: 10.1093/braincomms/fcae264 (PMC11339711; doi:10.1093/braincomms/fcae264)
Supplement: fcae264_Supplementary_Data [file fcae264_supplementary_data.zip › Supplementary_Figure 1.pdf]

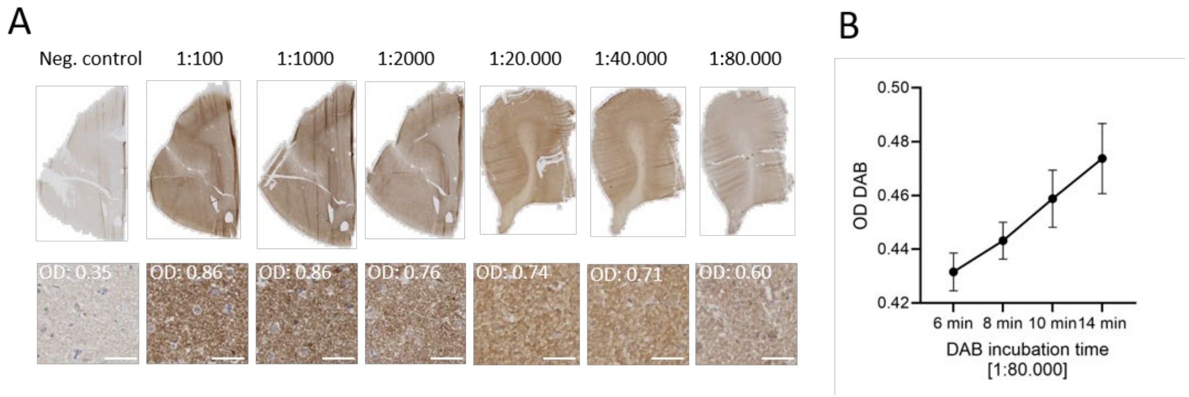

Supplemental figure 1: Serial dilutions of CD200 for optical density analysis, and optical density at [1:80,000] measured over DAB incubation time.
